# Association between diverse obesity indicators and sleep quality in elderly Chinese people: a National Study

**DOI:** 10.3389/fnut.2024.1459480

**Published:** 2024-10-11

**Authors:** Zhenzhen Liang, Wei Jin, Li Huang, Huajian Chen

**Affiliations:** ^1^Department of Epidemiology and Health Statistics, School of Public Health, Xinxiang Medical University, Xinxiang, Henan, China; ^2^Department of Vascular Surgery, The First Affiliated Hospital of Xinxiang Medical University, Weihui, Henan, China; ^3^School of Public Health, Wenzhou Medical University, Wenzhou, Zhejiang, China

**Keywords:** obesity indicators, sleep quality, elderly people, CLHLS, Chinese

## Abstract

**Background:**

The association between obesity indicators and sleep quality remains unclear among elderly Chinese people. Therefore, we aimed to assess this association by utilizing data from the Chinese Longitudinal Healthy Longevity Survey (CLHLS).

**Methods:**

A total of 10,505 participants aged 65 and above from the 2018 CLHLS were included. Calculate body mass index (BMI), waist circumference (WC), waist-to-height ratio (WHtR) and weight-adjusted-waist index (WWI) based on measured weight, height, and waist circumference. Based on BMI values, individuals were classified as underweight (<18.5 kg/m^2^), normal weight (18.5–23.9 kg/m^2^) and overweight or obesity (BMI ≥24 kg/m^2^). In the survey, sleep quality was rated in a 5-point format (“1 = very good,” “2 = good,” “3 = fair,” “4 = poor,” or “5 = very poor”), and we categorized “1” and “2” as good sleep quality and “3,” “4,” and “5” as poor sleep quality. Logistic regression models were used to evaluate odds ratios (ORs) and 95% confidence intervals (CIs), with subgroup analysis and restricted-cubic-spline (RCS) conducted.

**Results:**

The prevalence of poor sleep quality was 47.06%. There are significant differences in obesity indicators and other factors between the two groups of people with good sleep and poor sleep. After adjusting for potential confounding factors (including demographics, socioeconomic status, lifestyle behaviors, health-related issues and activities of daily living), our analyses revealed significant negative associations of BMI [OR 0.96 (95% CI 0.95–0.98)], WC [OR 0.99 (95% CI 0.98–0.99)] and WHtR [OR 0.18 (95% CI 0.09–0.35)] with poor sleep quality. RCS regression also showed that BMI, WC, WHtR and WWI were all strongly negatively correlated with poor sleep quality.

**Conclusions:**

In elderly Chinese people, overweight/obese elderly people may have a better sleep quality compared to elderly people with normal weight, while underweight elderly people are unfavorable for sleep quality.

## Introduction

Aging has become an important global public health challenge, and China is the country with the fastest aging population in the world (1). With the increasing elderly population, health and quality of life issues related to the elderly have become important social issues (2). It has been found that sleep problems are prevalent among elderly people (3), which has become one of the major problems affecting the health of elderly people (4).

In fact, sleep physiology changes with age, and with this comes a variety of sleep problems that lead to a general decline in sleep quality in elderly people (5). Decreased sleep quality can lead to serious and long-term illnesses (6, 7). Therefore, in recent years, the sleep problems of elderly people have received widespread attention.

Indeed, sleep disorders in elderly people should be viewed as a multifactorial geriatric health condition that requires consideration of multiple risk factors. Obesity rates are on the rise and obesity is a growing concern worldwide. A study in middle-aged adults showed that obesity affects sleep quality, one of the reasons being that increased visceral adipose tissue induces the secretion of inflammatory cytokines, which may disrupt the sleep wake rhythm (8). Another study of the elderly in China showed no significant correlation between Body mass index (BMI) and sleep quality (9). In addition, another study found that underweight was associated with poor sleep quality, whereas overweight or obesity was positively associated with good sleep quality (10). These studies used only BMI as a measure, which may explain part of the obesity paradox phenomenon. However, although BMI is the traditional parameter for assessing obesity, it mainly reflects the nutritional status and cannot display the distribution characteristics of fat and cannot differentiate between lean mass and fat mass, which has led many scholars to question its accuracy (11). Therefore, more research and reanalysis are still needed to assess the true obesity in the elderly people.

In recent years, researchers have proposed many new indicators of obesity. Waist circumference (WC) has been proposed as an alternative measure to indirectly assess visceral fat accumulation; Waist-to-height ratio (WHtR) is the ratio of WC to height, which has been reported the most cost-effective indicator for predicting hypertension in elderly people in China (12); Weight-adjusted waist index (WWI), which standardizes WC by body weight and is easy to measure, therefore, WWI can capture the benefits of WC and attenuate its correlation with BMI, primarily reflecting central obesity independent of body weight (13).

In this study, we used a national survey of the elderly people conducted in China to identify the association between four key obesity indicators (BMI, WC, WHtR, and WWI) and sleep quality. We focused on WC, WHtR and WWI, which could better reflect the true obesity status of elderly people than the traditional BMI. Through in-depth analysis of these alternative obesity indicators, our study can provide new insights into weight management in elderly people.

## Methods

### Study design and population

In this study, we selected data from the seventh wave (2017–2018) of the China Longitudinal Health and Longevity Survey (CLHLS). The CLHLS project is organized by the Center for Health Aging and Development at Peking University. This project conducted longitudinal surveys in 23 provinces of China in seven stages (2000, 2002, 2005, 2008–2009, 2011–2012, 2014, and 2017–2018). CLHLS adopts a multi-stage, disproportionate, and targeted random sampling method to ensure representativeness of the sample, with a focus on the population aged 60 and above. The participants included in this study are all elderly people aged 65 and above. After excluding incomplete questionnaires, the study included a sample size of 10,505. During the on-site investigation, all elderly people signed informed consent forms under the guidance of the researchers. The design of the CLHLS study was approved by the Duke University Campus Institutional Review Committee (Pro00062871) and the Peking University Biomedical Ethics Committee (IRB0001052-13074).

**Figure 1:**
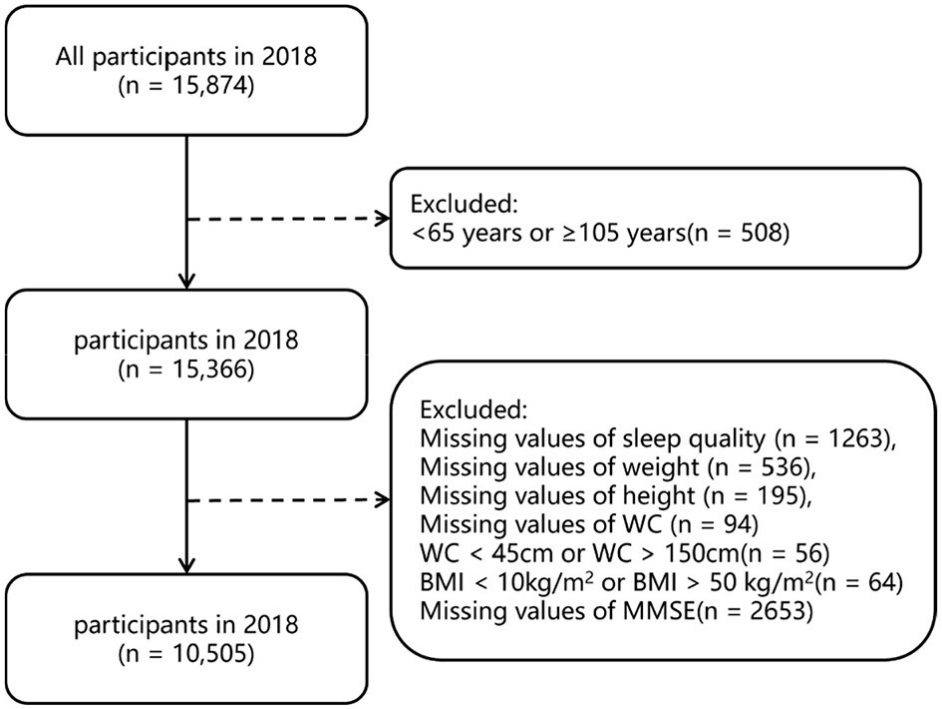
Flow diagram of how to select participants.

The survey included socio-demographic information, activities of daily living, and health-related questions. All data were obtained through face-to-face interviews. This study used the CLHLS 2018 wave to investigate the relationship between obesity and sleep quality in elderly people. Inclusion criteria for this study were: age ≥65 years; exclusion criteria were: (1) age >105 years, because there is no reliable information to verify their age, (2) samples with missing values (>10%) in the variable of interest. Missing values (<10%) were replaced using multiple imputation. The difference between the data with missing values before interpolation and the corresponding data after interpolation were compared, and the results showed no significant change between the two before and after interpolation (shown in Supplementary Table 1). Finally, a total of 10,505 participants were included in this study. Details of how participants were selected are shown in Figure 1.

### Exposure assessment

Height (cm) and weight (kg) were measured by measuring tape and weighing scale without shoes and heavy clothes. BMI is calculated using the following formula: BMI = weight (kg)/height (m)^2^. We categorized the participants into underweight (BMI <18.5 kg/m^2^), normal weight (BMI 18.5–23.9 kg/m^2^) and overweight or obesity (BMI ≥24 kg/m^2^) according to the Chinese BMI guideline (14). When measuring WC, participants were asked to be in an upright position with a calm exhalation, and a soft tape was used to measure around the midpoint of the line between the lower rib margin and the highest point of the iliac crest. Central obesity is defined as a waist circumference (WC) ≥80 cm in females or ≥85 cm in males (15). WHtR was calculated using the formula: WC (cm) divided by height (cm). WHtR was categorized into low WHtR (<0.5) and high WHtR (≥0.5) (16). WWI was calculated as WC (cm) divided by the square root of weight (kg), and low WWI was defined as WWI <11.25 cm/√kg while high WWI was defined as WWI ≥11.25 cm/√kg (16).

### Outcome definition

Sleep quality is evaluated through a single sleep quality scale, the details are as described in the reference (17). According to previous study (18), we convert sleep quality into a binary variable, i.e., good vs. poor. In the survey sleep quality was rated in a 5-point format (“1 = very good,” “2 = good,” “3 = fair,” “4 = poor,” or “5 = very poor”), and we categorized “1” and “2” as good sleep quality and “3,” “4,” and “5” as poor sleep quality.

### Covariates

The covariates were selected from standardized and structured questionnaire, which included demographics, socioeconomic status, lifestyle behaviors, health-related issues and activities of daily living (18, 19). Demographics included age, sex, occupation and marital status. Marital status was dichotomized into married and others. Socioeconomic status characteristics were measured by education level and residence. Lifestyles covered smoking status, alcohol drinking status and sleep duration. Health-related issues included hypertension, diabetes and cognitive impairment. Cognitive function refers to the psychological processes involved in knowledge acquisition, information manipulation, and reasoning, including perception, memory, learning, attention, decision-making, and language ability (20). Mini-Mental State Examination (MMSE) was utilized to assess cognitive function. MMSE score <24 was defined as cognitive impairment (15). The basic activities of daily living (BADL) and instrumental activities of daily living (IADL) were used to evaluate activities of daily living. The BADL consists of six items, such as bathing, dressing, toileting, and eating. Each item is scored from 1 to 3 (1 = don't need help; 2 = partially need help; 3 = need help). The BADL is defined as “needs help” when at least one item is selected as “partially needs help or needs help.” The IADL is about instrumental activities and consists of 8 items. IADL is defined as “dependent” when “partially or fully dependent” is selected for at least one item (18). In addition, according to the previous study (21), participants were categorized into <7 h (short), 7–8 h (moderate), and >8 h (long) nighttime sleep duration groups.

### Statistical analysis

All data statistical analyses were conducted using R (version 4.3.3). Continuous variables were tested for normality using the Kolmogorov-Smirnov test and reported as mean ± SD (standard deviation) if they conformed to a normal distribution or median (*Q*_1_, *Q*_3_) if they did not. Differences between groups were assessed by the Chi-square test (categorical data) and the *t*-test or Wilcoxon rank-sum test (continuous data). The association between sleep quality and obesity was then estimated using logistic regression with adjustment for confounding factors. We have developed three models to adjust for potential confounding factors. Model 1 adjusted for age and gender; Model 2 has adjusted health-related information based on Model 1; Model 3 has adjusted sleep time, cognitive impairment, IADL, and BADL based on Model 2. The relationship between BMI, WC, WHtR and WWI and sleep quality were evaluated using restricted-cubic-spline (RCS) regression analysis with 4 knots. Sensitivity analyses were performed to test the stability of the results by using another classification criteria. *P* values of <0.05 on both sides were considered statistically significant.

## Results

### Baseline demographics characteristics

A total of 10,505 participants were included in the analysis. Overall, the median (*Q*_1_, *Q*_3_) age was 82 (74, 91) years, and 47.2% were males. The median (*Q*_1_, *Q*_3_) of BMI, WC, WHtR and WWI were 22.3 (19.8, 25.0) kg/m^2^, 85 (78, 92) cm, 0.5 (0.5, 0.6), and 11.5 (10.8, 12.3) cm/√kg, respectively. The prevalence of poor sleep quality was 47.06%. Compared to good sleep quality individuals, poor sleep individuals tended to be female, no-formal education, non-professional work, not married, no smoking, no drinking, hypertension, diabetes, short sleep duration, dependence, cognitive impairment, underweight BMI, normal-weight WC and low WHtR (all *P* < 0.05).

Detailed information about the baseline demographic characteristics of all participants was illustrated in Table 1.

**Table 1:** Baseline characteristics of all participants grouped by sleep quality.

**Variables**	**Overall (*n* = 10,505)**	**Good sleep (*n* = 5,561)**	**Poor sleep (*n* = 4,944)**	***Z*/*χ^2^***	***P***
Age, years	82 (74, 91)	82 (74, 91)	82 (74, 90)	−0.275	0.784
**Sex**, ***n*** **(%)**				140.08	<0.001
Female	5,543 (52.8)	2,632 (47.3)	2,911 (58.9)		
Male	4,962 (47.2)	2,929 (52.7)	2,033 (41.1)		
**Education**, ***n*** **(%)**				50.07	<0.001
Formal education	5,925 (56.4)	3,316 (59.6)	2,609 (52.8)		
No formal education	4,580 (43.6)	2,245 (40.4)	2,335 (47.2)		
**Occupation**, ***n*** **(%)**				29.06	<0.001
Nonprofessional work	9,255 (88.1)	4,810 (86.5)	4,445 (89.9)		
Professional work	1,250 (11.9)	751 (13.5)	499 (10.1)		
**Residence**, ***n*** **(%)**				0.05	0.819
Rural	4,573 (43.5)	2,415 (43.4)	2,158 (43.6)		
Urban	5,932 (56.5)	3,146 (56.6)	2,786 (56.4)		
**Marital status**, ***n*** **(%)**				5.88	0.015
Married	5,125 (48.8)	2,775 (49.9)	2,350 (47.5)		
Other	5,380 (51.2)	2,786 (50.1)	2,594 (52.5)		
**Smoking**, ***n*** **(%)**				41.59	<0.001
No	8,752 (83.3)	4,510 (81.1)	4,242 (85.8)		
Yes	1,753 (16.7)	1,051 (18.9)	702 (14.2)		
**Drinking**, ***n*** **(%)**				82.54	<0.001
No	8,849 (84.2)	4,515 (81.2)	4,334 (87.7)		
Yes	1,656 (15.8)	1,046 (18.8)	610 (12.3)		
**Hypertension**, ***n*** **(%)**				12.79	<0.001
No	5,771 (54.9)	3,146 (56.6)	2,625 (53.1)	
Yes	4,734 (45.1)	2,415 (43.4)	2,319 (46.9)		
**Diabetes**, ***n*** **(%)**				5.4	0.02
No	9,432 (89.8)	5,029 (90.4)	4,403 (89.1)		
Yes	1,073 (10.2)	532 (9.6)	541 (10.9)		
**Sleep duration**, ***n*** **(%)**				2,216.06	<0.001
Long	2,567 (24.4)	1,988 (35.7)	579 (11.7)		
Moderate	3,961 (37.7)	2,614 (47)	1,347 (27.2)		
Short	3,977 (37.9)	959 (17.2)	3,018 (61)		
**BADL**, ***n*** **(%)**				1.07	0.301
Don't need help	9,006 (85.7)	4,786 (86.1)	4,220 (85.4)		
Need help	1,499 (14.3)	775 (13.9)	724 (14.6)		
**IADL**, ***n*** **(%)**				84.21	<0.001
Independence	4,391 (41.8)	2,556 (46)	1,835 (37.1)		
Dependence	6,114 (58.2)	3,005 (54)	3,109 (62.9)		
**MMSE**, ***n*** **(%)**				23.46	<0.001
Cognitive impairment	2,177 (20.7)	1,052 (18.9)	1,125 (22.8)		
Normal cognitive function	8,328 (79.3)	4,509 (81.1)	3,819 (77.2)		
**BMI (kg/m** ^**2**^ **)**	22.3 (19.8, 25)	22.5 (20, 25.2)	22.1 (19.6, 24.8)	−5.706	<0.001
**BMI**, ***n*** **(%)**				30.48	<0.001
Normal weight	5,482 (52.2)	2,884 (51.9)	2,598 (52.5)		
Overweight or obesity	3,540 (33.7)	1,975 (35.5)	1,565 (31.7)		
Underweight	1,483 (14.1)	702 (12.6)	781 (15.8)		
**WC (cm)**	85 (78, 92)	86 (79, 93)	84 (77, 91)	−7.899	<0.001
**WC**, ***n*** **(%)**				20.83	<0.001
Central obesity	6,593 (62.8)	3,603 (64.8)	2,990 (60.5)		
Not central obesity	3,912 (37.2)	1,958 (35.2)	1,954 (39.5)		
**WWI (cm/√kg)**	11.5 (10.8, 12.3)	11.5 (10.8, 12.2)	11.5 (10.8, 12.4)	−2.198	0.028
**WWI**, ***n*** **(%)**				1.01	0.314
High WWI	6,208 (59.1)	3,261 (58.6)	2,947 (59.6)		
Low WWI	4,297 (40.9)	2,300 (41.4)	1,997 (40.4)		
**WHtR**	0.5 (0.5, 0.6)	0.5 (0.5, 0.6)	0.5 (0.5, 0.6)	−2.598	0.009
**WHtR**, ***n*** **(%)**				8.93	0.003
High WHtR	8,021 (76.4)	4,311 (77.5)	3,710 (75)		
Low WHtR	2,484 (23.6)	1,250 (22.5)	1,234 (25)		

IADL, instrumental activities of daily living; BADL, basic activities of daily living; BMI, body mass index; WC, waist circumference; WHtR, waist-to-height ratio; WWI, weight-adjusted-waist index; MMSE, mini-mental state examination.

### Association of BMI, WC, WHtR and WWI with sleep quality

As shown in Table 2, we performed a multivariate logistic regression analysis to detect the association of BMI, WC, WHtR and WWI with sleep quality. When analyzed using continuous variables, in the fully adjusted model (model 3), the adjusted ORs for BMI, WC, WHtR, and WWI were 0.96 (95% CI: 0.95–0.98, *P* < 0.001), 0.99 (95% CI: 0.98–0.99, *P* < 0.001), 0.97 (95% CI: 0.93–1.01, *P* = 0.123) and 0.18 (95% CI: 0.09–0.35, *P* < 0.001), respectively. When analyzed by categorical variables, the adjust OR for underweight BMI group compared to the normal weight BMI group was 1.26 (95% CI: 1.10–1.44, *P* < 0.001), the adjust OR for the overweight or obesity BMI group compared to the normal weight BMI group was 0.81 (95% CI: 0.73–0.89, *P* < 0.001).

**Table 2:** Logistic regression analysis on the association of BMI, WC, WHtR and WWI with sleep quality.

**Exposure**	**Non-adjusted model**		**Model 1**		**Model 2**		**Model 3**	
	**OR (95% CI)**	***P***	**OR (95% CI)**	***P***	**OR (95% CI)**	***P***	**OR (95% CI)**	***P***
**BMI, kg/m** ^2^								
Per unit	0.97 (0.96, 0.98)	<0.001	0.97 (0.96, 0.98)	<0.001	0.97 (0.96, 0.98)	<0.001	0.96 (0.95, 0.98)	<0.001
Normal weight	Ref	–	–	–	–	–	–	–
Underweight	1.24 (1.10, 1.39)	<0.001	1.23 (1.09, 1.38)	<0.001	1.26 (1.12, 1.42)	<0.001	1.26 (1.10, 1.44)	<0.001
Overweight or obesity	0.88 (0.81, 0.96)	0.003	0.86 (0.79, 0.94)	<0.001	0.83 (0.76, 0.91)	<0.001	0.81 (0.73, 0.89)	<0.001
**WC, cm**								
Per unit	0.99 (0.98, 0.99)	<0.001	0.99 (0.98, 0.99)	<0.001	0.99 (0.98, 0.99)	<0.001	0.99 (0.98, 0.99)	<0.001
Not central obesity	Ref	–	–	–	–	–	–	–
Central obesity	0.83 (0.77, 0.9)	<0.001	0.77 (0.71, 0.84)	<0.001	0.76 (0.7, 0.82)	<0.001	0.75 (0.68, 0.82)	<0.001
**WWI, cm/**√**kg**								
Per unit	1.03 (1.00, 1.06)	0.047	0.98 (0.95, 1.01)	0.219	0.98 (0.94, 1.01)	0.148	0.97 (0.93, 1.01)	0.123
Low WWI	Ref	–	–	–	–	–	–	–
High WWI	1.04 (0.96, 1.13)	0.314	0.93 (0.86, 1.01)	0.096	0.92 (0.85, 1)	0.062	0.97 (0.93, 1.01)	0.123
**WHtR**								
Per unit	0.55 (0.32, 0.94)	0.03	0.25 (0.14, 0.43)	<0.001	0.2 (0.11, 0.36)	<0.001	0.18 (0.09, 0.35)	<0.001
Low WHtR	Ref	–	–	–	–	–	–	–
High WHtR	0.87 (0.8, 0.95)	0.003	0.81 (0.74, 0.89)	<0.001	0.79 (0.72, 0.87)	<0.001	0.8 (0.72, 0.89)	<0.001

Model 1 adjusted for age and gender; Model 2 adjusted health-related information based on Model 1; Model 3 adjusted sleep time, cognitive impairment, IADL, and BADL based on Model 2.

The adjust OR for the central obesity WC group compared to people without central obesity was 0.75 (95% CI: 0.68–0.82, *P* < 0.001). The adjust OR for high WHtR compared with the low WHtR was 0.80 (95% CI: 0.72–0.89, *P* < 0.001).

**Figure 2:**
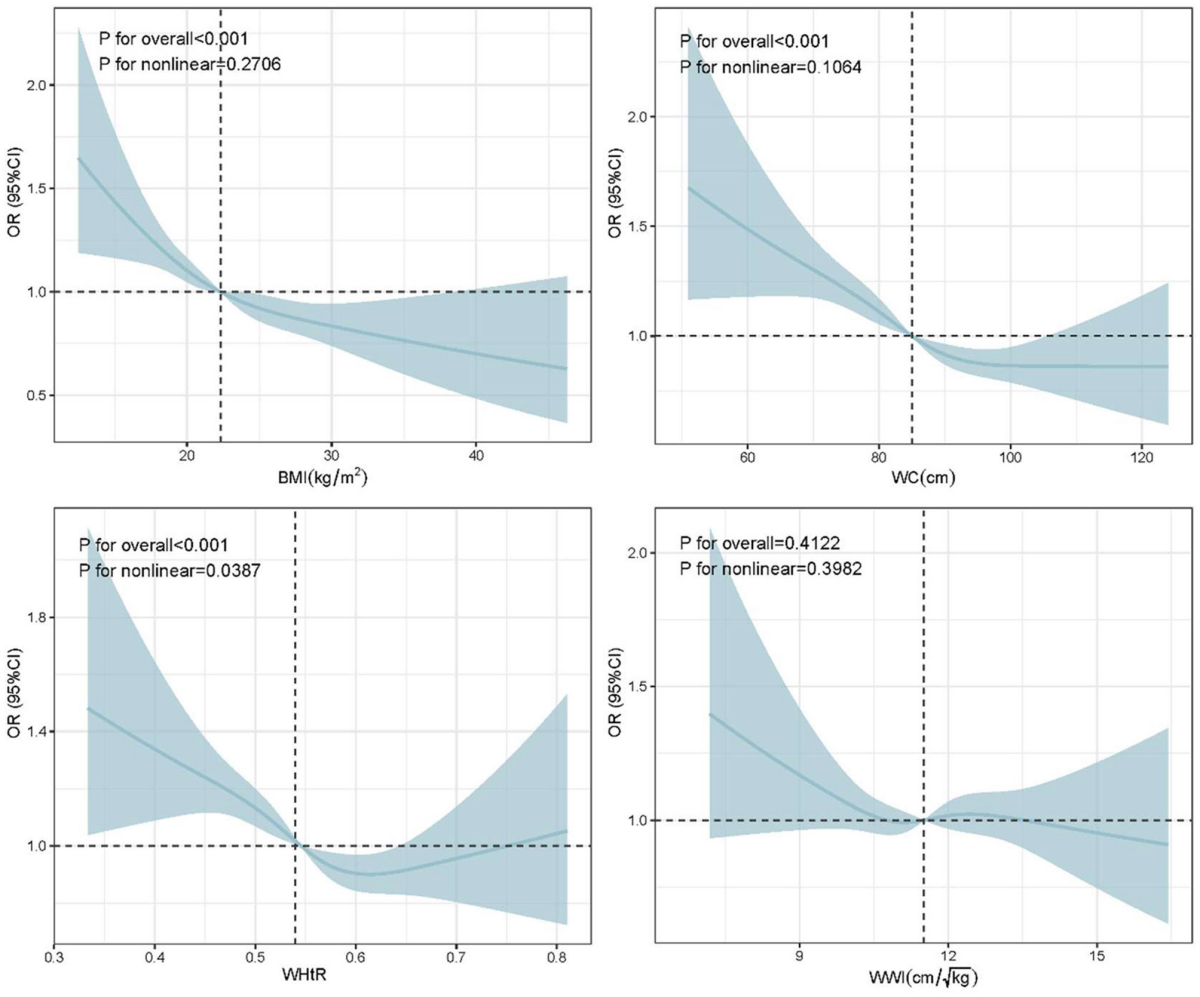
The RCS curves of the association between BMI, WC, WHtR and WWI and sleep quality. RCS regression was adjusted based on age, sex and sleep duration. RCS, restricted cubic spline.

We further evaluated the relationship between BMI, WC, WHtR and WWI and sleep quality using RCS analyses. And the RCS regression was adjusted based on age, sex and sleep duration. The results showed a strong negative association of BMI (*P*_overall_ <0.001, *P*_non−*linearity*_ = 0.271) and WC (*P*_overall_ <0.001, *P*_non−*linearity*_ = 0.106) with the risk of poor sleep quality; and there was an “L” shaped non-linear negative correlation between WHtR and poor sleep quality (*P*_overall_ <0.001, *P*_non−*linearity*_ = 0.039; Figure 2). Specifically, the risk of poor sleep quality reduced with rising in BMI and WC. The nonlinear curve showed that the curve leveled off after a WHtR of about 0.54, suggesting that the optimal WHtR for older people may be around overweight or mildly obese.

### Sensitivity analysis

Based on the classification criteria of BMI by Committee on Diet and Health (22), we further conducted a sensitivity analysis to test the stability of the results. As shown in Supplementary Table 2, the results revealed no substantial change when the classification criteria for BMI were changed, demonstrating the stability and reliability of the results generated by logistical regression analysis.

### Subgroup analyses

**Figure 3:**
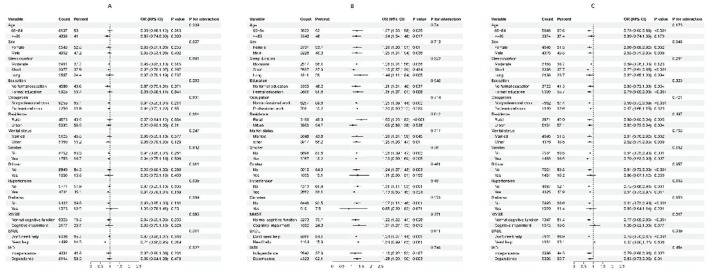
Subgroups analyses for the association between WWI and poor sleep quality **(A)**, the association between BMI and poor sleep quality in the under-weight BMI group and normal weight BMI group **(B)** and the association between BMI and poor sleep quality in the overweight or obesity BMI group and normal weight BMI group **(C)**. Analyses were stratified for sex (male and female), age (65–84 and ≥85 years), sleep duration (long, moderate and short), education (formal education and no formal education), occupation (nonprofessional work and professional work), residence (rural and urban), marital status (married and other), smoking status (smoker and non-smoker), alcohol consumption (drinker and non-drinker), diabetes (yes and no), hypertension (yes and no), BADL (“don't need help” and “need help”), IADL (independence and dependence) and MMSE (“cognitive impairment” and “normal cognitive function”).

Subgroup analysis was performed by age, sex, sleep duration, education, occupation, residence, marital status, smoking, drinking, hypertension, diabetes, cognitive impairment, BADL and IADL to explore whether the association between BMI, WC, WHtR and WWI and sleep quality remained stable across different subgroups. We found that none of the covariates significantly modified the association between WC or WHtR at the risk of poor sleep quality (All *P* for interaction >0.05; Supplementary Figures 1A, B). But the association between WWI and poor sleep quality were more pronounced in the oldest-old people (≥85 years old; *P* for interaction = 0.039) and the BADL disability (those who need help) people (*P* for interaction = 0.011; Figure 3A). In addition, compared with normal weight BMI group, in the under-weight BMI group, the adjusted OR was 1.50 (95% CI: 1.23–1.82, *P* < 0.001) for the variable of elderly people who live in the rural (Figure 3B). Moreover, compared with normal weight BMI group, in the overweight or obesity BMI group, the adjusted OR was 0.77 (95% CI: 0.69–0.86, *P* < 0.001) for the variable of normal cognitive function and 0.78 (95% CI: 0.70–0.87, *P* < 0.001) for the variable of good BADL (don't need help; Figure 3C).

## Discussion

Sleep quality is an important issue in the lives of elderly people, and our study systematically analyzed the relationship between various obesity indicators, including BMI, WC, WWI, and WHtR, and sleep quality using data from the CLHLS. Together, these indicators provide a comprehensive assessment of obesity from different bodily perspectives, and we employed multivariate logistic regression, sensitivity analysis, subgroup analysis, and restricted-cubic-spline curves to explore this complex relationship.

Our analysis revealed a 47.06% incidence of poor sleep quality among elderly people in China, indicating that nearly half of the elderly population suffers from sleep disturbances. This finding aligns with previous reports, which estimated the prevalence of sleep disorders in elderly populations to range between 10.4% and 62.1% (23). Our results also showed that sleep quality in females is worse than in males, consistent with many previous studies (24, 25). Moreover, elderly individuals with chronic diseases such as hypertension and diabetes were more likely to experience poor sleep quality, corroborating earlier research that linked poor sleep with these conditions (26–29). The reasons may be diabetes can lead to obstructive sleep apnea and nocturia, while hypertension-related sleep disturbances are associated with elevated nighttime blood pressure (3, 30–32). We also considered the impact of socioeconomic factors such as marital status on sleep, but they were not the primary focus of our study. Married elderly individuals appeared to have better sleep quality, possibly due to emotional support from their spouses, whereas widowed or divorced elderly may face emotional stress and loneliness, which could negatively affect their sleep (33–36). In addition, cognitive decline was associated with poor sleep quality, potentially due to its impact on brain regions responsible for sleep regulation, such as the thalamus and pineal gland (37). Cognitive impairment is often accompanied by psychological issues like depression and anxiety, further contributing to disturbed sleep (38).

As for obesity indicators and sleep quality, our results showed that after adjusting for other covariates, the adjust OR for the overweight or obesity BMI group compared to the normal weight BMI group was 0.81 (95% CI: 0.73–0.89); the adjust OR for the central obesity WC group compared to people without central obesity was 0.75 (95% CI: 0.68–0.82) and the adjust OR for high WHtR compared with the low WHtR was 0.80 (95% CI: 0.72–0.89). All the results indicated that obesity may have a protective effect on sleep quality in elderly people. Traditionally, obesity has been considered a risk factor for various sleep disorders and obesity contributes to sleep disorders through multiple mechanisms, including increased upper airway resistance, altered respiratory mechanics, and systemic inflammation (39). Conversely, sleep disorders exacerbate obesity by disrupting metabolic and hormonal regulation (40). So, there is a strong bidirectional causal relationship between obesity and poor sleep quality. However, our results contradict the traditional views. Our research demonstrates that obesity is a protective factor for sleep quality in the elderly Chinese people, and obese elderly people are less likely to develop sleep disorders compared to normal weight elderly people. This counterintuitive concept warrants a closer examination of the mechanisms and clinical implications involved. But we have not found any evidence that has been reported to support our viewpoint. We considered the possible reasons from the following points: (1) Obesity provides a metabolic reserve that is beneficial during periods of illness or stress. Increasing fat storage may help maintain energy balance and support physiological elasticity, potentially stabilizing sleep patterns during acute stress attacks; (2) Adipose tissue secretes various inflammatory and metabolic factors that affect sleep regulation (41), such as leptin, which can reduce appetite and promote satiety and is related to sleep quality (42). Higher levels of leptin in obese individuals may contribute to more stable sleep patterns; (3) Obesity can affect body temperature regulation, which is crucial for sleep (43). The adipose tissue can help maintain core body temperature, potentially promoting better sleep continuity and reducing nocturnal arousal, thereby improving sleep quality.

Although there have been no studies reporting that obesity is a protective factor for sleep quality in the elderly people, several studies on the “obesity paradox” have given us confidence in our data. The “obesity paradox” refers to the lower mortality rate in the group with higher BMI and the higher mortality rate in the group with underweight. The reason may be that better nutritional intake and excessive fat reserves can provide energy, calories, and the ability to resist hunger and malnutrition, which in turn has a protective effect on disease survivors (44, 45).

However, it is worth noting that although our results support obesity as a protective effect on sleep quality, this relationship is complex and may be not universally applicable. Obesity remains an important risk factor for many sleep disorders, and its protective effect may be limited to subgroups of the elderly people. At present, research on sleep quality management for the elderly people population in China needs to be strengthened, and more attention should be paid to the issue of sleep disorders to improve the sleep quality of the elderly people. Future research should focus on longitudinal studies to track the interaction between obesity and sleep quality in elderly people. In addition, exploring the genetic and environmental factors that regulate this relationship is crucial for a comprehensive understanding of the potential mechanisms between obesity and sleep quality in the elderly people, and for developing targeted intervention measures.

### Strengths and limitations

The strength of this study lies in its use of nationally representative data from the elderly Chinese people, providing a strong basis for verifying the association between obesity and sleep quality. This study conducted comprehensive covariate adjustments and subgroup analyses to minimize potential confounders that could interfere with the results. Adjusting for various covariates allowed us to include major potential confounders and to better explain the association between obesity and sleep disorders. Subgroup analysis, focusing on demographics, socioeconomic status, lifestyle behaviors, health-related issues and activities of daily living, adds practical guiding significance to the findings. However, this study has certain limitations. First, as a cross-sectional study, sleep quality and the presence of obesity were measured at the same time, therefore, it cannot establish a causal relationship, so the findings can only be interpreted statistically (18). Longitudinal data may be needed to further explore this relationship. Secondly, the ORs obtained using logistic regression may be biased at high prevalence rates, which can lead to overestimation of ORs. However, considering the design and sample characteristics of the current study, we opted for logistic regression. This method has been widely used in similar studies, and its feasibility for higher prevalence has been demonstrated by appropriately adjusting and interpreting the results (18, 46–49). Furthermore, in our study, we mitigated this problem by controlling for confounders as much as possible. Nevertheless, future studies should aim to validate these findings using other appropriate statistical methods to ensure the robustness of the results under different analytical frameworks. Thirdly, the study population was representative of elderly people in China, so caution should be exercised in generalizing existing findings to other age groups, regions, or ethnicities. Lastly, despite extensive adjustments for confounders, there may be some potential confounding factors that were not controlled for such as health conditions, medication use, dietary habits, and others. However, due to data set limitations, we were unable to obtain a comprehensive picture of other confounding factors that affect sleep quality. Therefore, more relevant studies are needed to provide evidence for the current findings.

Our findings provide insights and motivation for further research in this area. Additional cohort studies or intervention trials, or modifications to BMI standards for elderly people, are necessary to recognize the potential relationship between obesity and sleep quality in elderly people.

## Conclusions

In elderly Chinese people, overweight/obese elderly people may have a better sleep quality compared to elderly people with normal weight, while underweight elderly people are unfavorable for sleep quality.

## Data Availability

The original contributions presented in the study are included in the article/Supplementary material, further inquiries can be directed to the corresponding author.
