# Supplementary material for: Association between diverse obesity indicators and sleep quality in elderly Chinese people: a National Study
Source: Front Nutr. 2024 Oct 11;11:1459480. doi: 10.3389/fnut.2024.1459480 (PMC11512449; doi:10.3389/fnut.2024.1459480)
Supplement: Supplementary file 1 [file Data_Sheet_1.docx]

**Supplementary Table 1** Difference between the data with missing values before interpolation and the corresponding data after interpolation

|  | After imputation | Before imputation | *χ^2^* | *P* |
| --- | --- | --- | --- | --- |
| Total | 10505 | 10505 |  |  |
| BADL, n (%) |  |  | 0.55 | 0.458 |
| Don't need help | 9006 (85.7) | 8640 (85.4) |  |  |
| Need help | 1499 (14.3) | 1481 (14.6) |  |  |
| IADL, n (%) |  |  | 0.00 | 0.968 |
| Independence | 4391 (41.8) | 4360 (41.8) |  |  |
| Dependence | 6114 (58.2) | 6064 (58.2) |  |  |
| Education, n (%) |  |  | 0.00 | 0.968 |
| Formal education | 5925 (56.4) | 5841 (56.4) |  |  |
| No formal education | 4580 (43.6) | 4510 (43.6) |  |  |
| Job, n (%) |  |  | 0.93 | 0.335 |
| Nonprofessional work | 9255 (88.1) | 8839 (87.7) |  |  |
| Professional work | 1250 (11.9) | 1244 (12.3) |  |  |
| Sleep duration, n (%) |  |  | 0.00 | 0.998 |
| Long | 2567 (24.4) | 2548 (24.4) |  |  |
| Moderate | 3961 (37.7) | 3940 (37.7) |  |  |
| Short | 3977 (37.9) | 3956 (37.9) |  |  |
| Marital status, n (%) |  |  | 0.01 | 0.939 |
| Married | 5125 (48.8) | 5090 (48.8) |  |  |
| Other | 5380 (51.2) | 5332 (51.2) |  |  |
| Smoking, n (%) |  |  | 0.05 | 0.830 |
| No | 8752 (83.3) | 8663 (83.2) |  |  |
| Yes | 1753 (16.7) | 1749 (16.8) |  |  |
| Drinking, n (%) |  |  | 0.16 | 0.686 |
| No | 8849 (84.2) | 8709 (84) |  |  |
| Yes | 1656 (15.8) | 1655 (16) |  |  |
| Hypertension, n (%) |  |  | 0.83 | 0.362 |
| No | 5771 (54.9) | 5397 (54.3) |  |  |
| Yes | 4734 (45.1) | 4542 (45.7) |  |  |
| Diabetes, n (%) |  |  | 3.44 | 0.064 |
| No | 9432 (89.8) | 8518 (89) |  |  |
| Yes | 1073 (10.2) | 1055 (11) |  |  |

**Supplementary Table 2** Logistic regression analysis on the association of BMI with sleep quality based on the classification criteria of BMI by Committee on Diet and Health

| Exposure | | Non-adjusted model | |  | Model 1 | |  | Model 2 | |  | Model 3 | |
| --- | --- | --- | --- | --- | --- | --- | --- | --- | --- | --- | --- | --- |
|  |  | OR (95%CI) | *P* |  | OR (95%CI) | *P* |  | OR (95%CI) | *P* |  | OR (95%CI) | *P* |
| BMI, kg/m^2^ | Normal weight | Ref | - |  | Ref | - |  | Ref | - |  | Ref | - |
|  | Underweight | 1.16 (1.06,1.26) | < 0.001 |  | 1.17 (1.07,1.28) | < 0.001 |  | 1.20 (1.10,1.32) | < 0.001 |  | 1.23 (1.11,1.36) | < 0.001 |
|  | Overweight or obesity | 0.82 (0.69,0.98) | 0.032 |  | 0.80 (0.67,0.95) | 0.013 |  | 0.78 (0.65,0.93) | 0.006 |  | 0.74 (0.61,0.91) | 0.004 |

Model 1 adjusted for age and gender; Model 2 has adjusted health-related information based on Model 1; Model 3 has adjusted sleep time, cognitive impairment, IADL, and BADL based on Model 2.

In people aged 65, underweight: BMI < 23 kg/m^2^, normal weight 23–28 kg/m^2^, and overweight > 28 kg/m^2^; and in people aged 66 or older underweight: BMI < 24 kg/m^2^, normal weight 24–29 kg/m^2^, and overweight > 29 kg/m^2^.


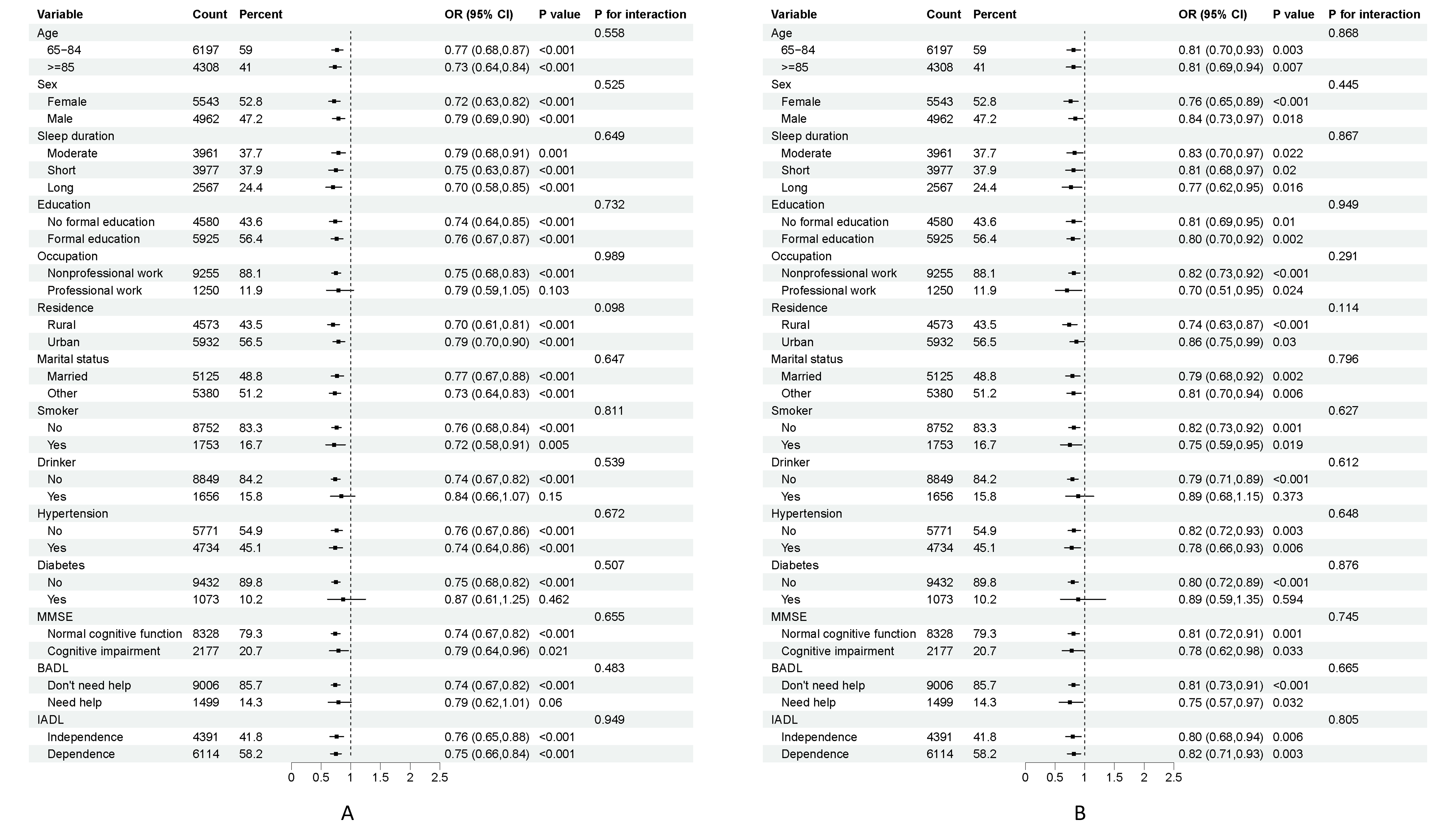


**Supplementary Figure 1** Subgroups analyses for the association between WC (A), WHtR (B) and poor sleep quality.
